# Pregnancy and obstetric outcomes of dichorionic and trichorionic triamniotic triplet pregnancy with multifetal pregnancy reduction: a retrospective analysis study

**DOI:** 10.1186/s12884-022-04617-y

**Published:** 2022-04-05

**Authors:** Shuhua Liu, Guanjian Li, Chao Wang, Ping Zhou, Zhaolian Wei, Bing Song

**Affiliations:** 1 Reproductive Medicine Center, Department of Obstetrics and Gynecology, the First Affiliated Hospital of Anhui Medical University, Hefei, 230032 China; 2 Department of Obstetrics and Gynecology, Anhui Province Maternity and Child Health Hospital, Hefei, 230000 China; 3 NHC Key Laboratory of Study on Abnormal Gametes and Reproductive Tract, Anhui Medical University, Hefei, 230032 China; 4 Ministry of Education Key Laboratory of Population Health Across Life Cycle, Anhui Medical University, Hefei, 230032 China; 5 Anhui Province Key Laboratory of Reproductive Health and Genetics, Hefei, 230032 China

**Keywords:** Multifetal pregnancy reduction, Obstetric outcomes, Dichorionic triamniotic, Assisted reproductive technology

## Abstract

**Background:**

It is generally beneficial for triplet gestation or high-order multiple pregnancies to operate multifetal pregnancy reduction (MFPR) after assisted reproductive techniques. However, data on pregnancy outcomes is lacking regarding dichorionic triamniotic (DCTA) and trichorionic triplets (TCTA) pregnancy.

**Method:**

This research analyzes the difference between 128 DCTA and 179 TCTA pregnancies with or without MFPR after in vitro fertilization/intracytoplasmic sperm injection cycles between January 2015 and June 2020. The subdivided subgroups of the two groups are reduction to singleton, reduction to dichorionic twins, and expectant management groups. We also compare the pregnancy and obstetric outcomes between 2104 dichorionic twins and 122 monochorionic twins.

**Result:**

The research subgroups were DCTA to monochorionic singleton pregnancies (*n* = 76), DCTA to dichorionic twin pregnancies (*n* = 18), DCTA-expectant management (*n* = 34), TCTA to monochorionic singleton pregnancies (*n* = 31), TCTA to dichorionic twin pregnancies (*n* = 130), and TCTA-expectant management (*n* = 18). In DCTA-expectant management group, the complete miscarriage rate is dramatically higher, and the survival rate and the rate of take-home babies are lower. However, there was no difference between the rates of complete miscarriages, survival rates, and take-home babies in TCTA-expectant management group. But the complete miscarriage rate of DCTA-expectant management was obviously higher than that of TCTA-expectant management group (29.41 vs. 5.56%, *p* = 0.044). For obstetric outcomes, MFPR to singleton group had higher gestational week and average birth weight, but lower premature delivery, gestational hypertension rates and low birth weight in both DCTA and TCTA pregnancy groups (all *p* < 0.05). DCTA to monochorionic singleton had the lowest incidence of gestational diabetes, whereas The subdivided subgroups of TCTA had no significant difference in the incidence of gestational diabetes. Monochorionic twins have higher rates of complete, early, and late miscarriage, premature delivery, and late premature delivery, and lower survival rate (*p* < 0.05).

**Conclusion:**

MFPR could improve gestational week and average birth weight, reducing premature delivery, LBW, and gestational hypertension rates in DCTA and TCTA pregnancies. Monochorionic twins have worse pregnancy and obstetric outcomes. MFPR to singleton is preferable recommended in the pregnancy and obstetric management of complex triplets with monochorionic pair.

## Introduction

Over the past decades, the incidence of multiple pregnancies has obviously increased [1], mainly owing to the wide application of assisted reproductive techniques (ART) [2]. Controlled ovarian hyperstimulation is involved in ART for the initial step, which is used to obtain multiple embryos [3], and multiple embryo transplants are used to maximize pregnancy rates in ART [4, 5]. Our previous study showed that the young mother’s age, high-quality embryos are risk factors of monochorionic-diamniotic (MC-DA) twin in assisted reproduction [6]. With the increase in the number of embryos, the incidence of fetal and maternal complications is also elevated. These risks include gestational diabetes mellitus, gestational hypertension and medical and surgical conditions aggravation. Meanwhile, there are higher risks of miscarriage, embryonic growth restriction, premature delivery, and the complications of fetal respiratory diseases and cerebral palsy caused by premature delivery [7, 8]. In addition, a monochorionic pregnancy is associated with specific complications caused by vascular anastomoses in the common placenta, which impact infant and maternal morbidity and mortality [9]. Monochorionic twins have attracted extensive attention as a result of the associated complications, for example twin anemia-polycythemia sequence (TAPS), twin-to-twin transfusion syndrome (TTTS), and selective intrauterine growth restriction (SIGR).

Multifetal pregnancy reduction (MFPR) is extensively used to decrease maternal and fetal risks to raise the likelihood of good pregnancy outcomes [10, 11], but no agreed optimal strategy exists. Some scholars think that reduction to single can achieve the most perfect pregnancy and obstetric outcomes [12, 13], however other studies have found that the rate of miscarriage could be increased after early MFPR [14, 15]. In addition, there are few published reports concerning the expectant management of DCTA pregnancies, in which a pair of monochorionic diamniotic twins use a placenta at the same time.

This study aimed to analyze the differences between MFPR and expectant management in DCTA and TCTA pregnancies, and analyze the differences between monochorionic and dichorionic twins’ pregnancies.

## Materials and methods

We retrospectively analyzed 128 DCTA cases, 179 TCTA cases, 2104 dichorionic twin cases, and 122 monochorionic twin cases from January 2015 to June 2020 at the Reproductive Medicine Center, Department of Obstetrics and Gynecology, the First Affiliated Hospital of Anhui Medical University. The subdivided subgroups of the two groups are DCTA reduction to monochorionic singleton (*n* = 76), DCTA reduction to dichorionic-diamniotic (DCDA) twin (*n* = 18) and DCTA-expectant management (*n* = 34) groups, and TCTA reduction to monochorionic singleton (*n* = 31), TCTA reduction to dichorionic twin (*n* = 130), and TCTA-expectant management (*n* = 18) groups.

All of MFPRs were performed 6–8 weeks after embryo transplant (ET). Experienced doctors in our Reproductive Medicine Center carried out these operations, which involve the puncture and aspiration of selected embryonic parts without administering any medications, under transvaginal ultrasound guidance. Pregnancies were diagnosed by transvaginal ultrasound to determine the number of fetal and monochorionic or dichorionic pregnancies according to the ultrasound presence of the “T sign” or “lambda sign”. Couples were informed about the disadvantages of triplet or high-order multiple pregnancies. We finally decided to perform the operation based on the couples’ intention, the situation, and the fetus’s condition. Pregnant mothers had been protected from infection by administering antibiotics for 1 week preoperatively, and the fetuses were inspected in utero on the 1st and 5th day postoperatively.

### Statistical analysis

Statistical analysis were performed using the SPSS 22.0 software package (SPSS Inc., Chicago, IL). Because the data were normally distributed, continuous variables are expressed as mean ± standard deviation (SD). Between-group differences were evaluated using a t-test, and the fisher’s exact test or the chi-squared test were used to analyze the difference between percentages. The threshold of the data discrepancies was set at *P* < 0.05.

## Results

### The maternal demographics and clinical characteristics in TCTA and DCTA pregnancies

The clinical features of DCTA and TCTA groups included in this study are listed in Table 1 and Table 2. There were no statistically significant differences of the maternal age between the subgroups. Body mass index (BMI), the interval between transplantation and MFPR, duration and type of infertility, frozen embryo transplant (FET), and insemination methods were also matched in both groups with no significant differences(*P* > 0.05).

**Table 1:** Maternal demographics and clinical characteristics in DCTA pregnancy

	DCTA reduction to twin (*n* = 18)	DCTA reduction to singleton (*n* = 76)	DCTA-expectant management (*n* = 34)	*P* value
Clinical characteristic				
Maternal age (years)	30.33 ± 4.37	29.47 ± 4.07	29.21 ± 4.35	0.645
Interval between transplantation and MFPR (days)	41.83 ± 6.38	40.08 ± 6.19		0.285
BMI (kg/m^2^)	21.09 ± 2.70	22.39 ± 2.93	21.05 ± 2.10	0.069
Duration of infertility (years)	3.53 ± 2.05	3.46 ± 2.56	3.41 ± 2.16	0.986
Infertility type				
Primary, n (%)	(14/18) 77.78	(42/76) 55.26	(17/34) 50.00	
Secondary, n (%)	4	34	17	0.139
FET (N)				
not-used (%)	(7/18) 38.89	(26/76) 34.21	(9/34) 26.47	
used (%)	11	50	25	0.610
Insemination methods				
ICSI, n (%)	(7/18) 61.70	(29/76) 63.64	(11/34) 47.83	
IVF, n (%)	11	47	23	0.826

No significant difference was found between the three sets of data in the DCTA group

**Table 2:** Comparison of the maternal demographics and clinical characteristics in TCTA pregnancies

	TCTA reduction to single (*n* = 31)	TCTA reduction to twin (*n* = 130)	TCTA-expectant management (*n* = 18)	*P* value
Clinical characteristic				
Maternal age (years)	32.57 ± 3.77	32.20 ± 4.57	32.00 ± 3.48	0.634
Interval between transplantation and MFPR (days)	38.86 ± 3.34	37.52 ± 5.19		0.707
BMI (kg/m^2^)	21.41 ± 1.89	22.31 ± 2.92	22.14 ± 3.16	0.235
Duration of infertility (years)	3.94 ± 1.67	4.06 ± 2.51	5.11 ± 3.09	0.073
Infertility type				
Primary, n (%)	(19/31)61.29	(79/130)60.77	(9/18)50.00	
Secondary, n (%)	12	51	9	0.671
FET (N)				
not-used (%)	(7/31)22.58	(26/130)20.00	(6/18)33.33	
Used (%)	24	104	12	0.435
Insemination methods				
ICSI, n (%)	(7/31) 22.58	(35/130) 26.92	(3/18) 16.67	
IVF, n (%)	24	95	15	0.602

No significant difference was found between the three sets of data in the TCTA group

### Pregnancy and obstetric outcomes in DCTA and TCTA pregnancies

DCTA reduction to singleton group had lower rates of complete miscarriage, early miscarriage, higher rates of survival and take-home babies than DCTA- expectant management group (Table 3). Furthermore, the obstetric outcomes were better in DCTA reduction to singleton pregnancy group than DCTA reduction to twin and DCTA-expectant management groups, which shows that DCTA reduction to singleton group had the longest gestational week and the highest average birth weight, while the rates of early miscarriage, premature delivery, late premature delivery, gestational diabetes mellitus, and LBW were the lowest. In addition, the rate of late miscarriage, early premature delivery, survival rate, gestational hypertension were all lower in DCTA to singleton group than in DCTA expectant management group. However, cesarean section rate, the rate of VLBW, and the percentage of boys were not significant different between three DCTA subgroups.

**Table 3:** Pregnancy and obstetric outcomes in DCTA pregnancy

	DCTA reduction to twin (*n* = 18)	DCTA reduction to singleton (*n* = 76)	DCTA-expectant management (*n* = 34)	*P* value
pregnancy outcomes				
gestational week (weeks)	37.19 ± 2.90^a^	38.85 ± 1.69^a,b^	35.33 ± 2.35	***p*** **< 0.001**
Average birth weight (g)	2734.09 ± 615.55^a^	3276.49 ± 515.64^a,b^	2232.65 ± 603.53	***p*** **< 0.001**
Complete miscarriage rate (%)	(2/18) 11.11	(2/76) 2.63^a^	(10/34) 29.41	***P*** **< 0.001**
Early miscarriage rate(< 12 weeks) (%)	(1/18) 5.55	(0/76) 0.00^a,b^	(5/34) 14.71	***p*** **= 0.003**
Late miscarriage rate (12–28 weeks)	(1/18) 5.55	(2/76) 2.63^a^	(5/34) 14.71	***p*** **= 0.042**
Premature delivery rate (%)	(7/16)43.75	(7/74) 9.46^a,b^	(16/24) 66.67	***p*** **< 0.001**
Early premature delivery (28–34 weeks) (%)	(1/16) 6.25	(3/74) 4.05^a^	(5/16) 31.25	***p*** **= 0.006**
Late premature delivery (34-37 weeks) (%)	(6/16)37.5	(4/74) 5.41^a,b^	(11/16) 68.75	***p*** **< 0.001**
survival rate(%)	(16/18)88.89	(74/76) 97.37^a^	(24/34) 70.59	***p*** **< 0.001**
One survivor	10	74	6	/
Two survivors	6	–	11	/
Three survivors	–	–	7	/
Take baby home rate (%)	(16/18) 88.89	(74/76) 97.37^a^	(24/34) 70.59	***p*** **< 0.001**
Cesarean section rate (%)	(13/16) 81.25	(60/74) 81.08	(23/24) 95.83	*p* = 0.220
LBW < 2500 g (%)	(9/22) 40.91	(3/74) 4.05^a,b^	(24/49) 48.98	***p*** **< 0.001**
VLBW < 1500 g (%)	(1/22) 4.55	(1/74) 1.35	(3/49) 6.12	*p* = 0.358
Percentage of boys (%)	(10/12) 83.33	(42/74) 56.76	(26/49) 6.12	*p* = 0.161
obstetric outcomes				
Gestational hypertension (%)	(2/16) 12.5	(3/74) 4.05^a^	(6/24) 25	***p*** **= 0.007**
Gestational diabetes mellitus (%)	(3/16) 18.75	(1/74) 1.35^a,b^	(4/24) 16.67	***p*** **= 0.004**

^a^ Significant difference compared with the expectant management group

^b^ Significant difference compared with the DCTA to twin group

The premature delivery rate was highest in TCTA-expectant management group, but gestational week and average birth weight were lowest. However, complete miscarriage, survival rate, cesarean section, VLBW, and take-home baby rates or the percentage of boys were no significant difference between three TCTA subgroups (Table 4). The obstetric outcomes were similar in DCTA and TCTA pregnancies, and the MFPR to singleton group had the longest gestational week, highest average birth weight, and lowest rate of LBW. Conversely, the premature delivery and LBW rates were highest in DCTA-expectant management and TCTA-expectant management groups.

**Table 4:** Pregnancy and obstetric outcomes in TCTA pregnancy

	TCTA reduction to twin (*n* = 130)	TCTA reduction to singleton (*n* = 31)	TCTA-expectant management(*n* = 18)	*P* value
pregnancy outcomes				
gestational week (weeks)	35.99 ± 2.33^a^	38.00 ± 2.64^a,b^	34.64 ± 2.79	***p*** **< 0.001**
Average birth weight (g)	2567.66 ± 571.38^a^	3105.38 ± 691.46^a,b^	2285.43 ± 613.71	***p*** **< 0.001**
Complete miscarriage rate (%)	(5/130) 3.85	(3/31) 9.68	(1/18) 5.56	*p* = 0.309
Early miscarriage rate(< 12 weeks) (%)	(1/130) 0.77	(2/31) 6.45	(1/18) 5.56	*p* = 0.063
Late miscarriage rate (12–28 weeks)	(4/130) 3.08	(1/31) 3.23	(0/18) 0.00	*p* = 1.000
Premature delivery rate	(60/125) 48.00^a^	(5/28) 17.86^a,b^	(15/17) 88.24	***p*** **< 0.001**
Early premature delivery (28–34 weeks) (%)	(20/125) 16.00	(1/28) 3.57	(2/17) 11.76	*p* = 0.235
Late premature delivery (34–37 weeks) (%)	(40/125) 32.00^a^	(4/28) 14.29^a^	(13/17) 74.47	***p*** **< 0.001**
survival rate(%)	(124/130) 95.38	(28/31) 90.32	(17/18) 94.44	*p* = 0.482
One survivor	16	28	2	/
Two survivors	108	–	5	/
Three survivors	–	–	10	/
Take baby home rate (%)	(124/130) 95.38	(28/31) 90.32	(17/18) 94.44	*p* = 0.482
Cesarean section rate	(112/125) 89.6	(23/28) 82.14	(15/17) 88.24	*p* = 0.482
LBW < 2500 g (%)	(92/232) 39.66	(3/28)10.71^a,b^	(23/42) 54.76	***P*** **= 0.001**
VLBW < 1500 g (%)	(28/232) 12.07	(1/28) 3.57^a^	(6/42) 14.29	***p*** **= 0.359**
Percentage of boys (%)	(118/232)50.86	(14/28)50	(26/49)53.06	*P* = 0.432
obstetric outcomes				
Gestational hypertension (%)	(9/125)0.072	(0/28) 0^a^	(4/17)23.53	***P*** **= 0.020**
gestational diabetes mellitus (%)	(5/125) 4	(1/28)3.57	(3/17)17.65	*P* = 0.087

^a^Significant difference compared with the expectant management group

^b^Significant difference compared with the TCTA to twin group

### Comparison of pregnancy and obstetric outcomes in monochorionic and dichorionic twin pregnancies

We analyzed 2226 cases of twin pregnancies, including 122 cases of monochorionic and 2104 cases of dichorionic twins. The pregnancy and obstetric outcomes of the two groups were shown in Table 5. The results showed that monochorionic twin have higher rates of complete miscarriage (24.59 vs. 7.27%, *p* < 0.001), early miscarriage (13.93 vs. 2.04%, *p* < 0.001), late miscarriage (10.66 vs. 5.23%, *p* < 0.05), premature delivery (60.87 vs. 48.23%, *p* < 0.05), late premature delivery (50.00 vs. 36.90%, *p* < 0.05), and TTTS (3.28 vs. 0%, *p* < 0.001), but lower rates of survival rate (75.41 vs. 92.16%, *p* < 0.001), and multiple survival rates (61.48 vs. 78.33%, *p* < 0.001) than dichorionic twins.

**Table 5:** Monochorionic and dichorionic twin pregnancies comparison

	Dichorionic twins (*n* = 2104)	Monochorionic twins (*n* = 122)	*P* value
Clinical characteristic			
Maternal age (years)	29.49 ± 3.69	29.58 ± 4.17	0.797
gestational week (weeks)	36.20 ± 2.27	36.05 ± 2.30	0.542
Average birth weight (g)	2612.53 ± 523.61	2566.37 ± 507.27	0.263
Proportion of primary infertility (%)	(1250/2104)59.41	(73/122)59.84	0.926
Fresh cycle ratio (%)	(632/2104)30.04	(34/122)27.87	0.611
pregnancy outcomes			
Complete miscarriage rate (%)	(153/2104)7.27	(30/122)24.59	***p*** **< 0.001**
Early miscarriage rate(< 12 weeks) (%)	(43/2104)2.04	(17/122)13.93	***P*** **< 0.001**
Late miscarriage rate (12–28 weeks)	(110/2104)5.23	(13/122)10.66	**0.011**
Premature delivery rate (%)	(941/1951)48.23	(56/92)60.87	**0.018**
Early premature delivery (28–34 weeks) (%)	(221/1951)11.33	(10/92)10.87	0.892
Late premature delivery (34-37 weeks) (%)	(720/1951)36.90	(46/92)50.00	**0.011**
survival rate rate (%)	(1939/2104)92.16	(92/122)75.41	***p*** **< 0.001**
Singleton survival rate rate (%)	(291/2104)13.83	(14/122)11.48	0.462
Twin survival rate rate (%)	(1648/2104)78.33	(75/122)61.48	*p* < 0.001
Percentage of twins, one live, one stillbirth (%)	(0/2104)0	(3/122)2.46	*p* < 0.001
Double stillbirth (%)	(12/2104)0.57	(0/122)0.00	1.000
Cesarean section rate (%)	(1776/1951)91.03	(86/92)93.48	0.419
LBW rate (%) (< 2500 g)	(1293/3611)35.81	(69/170)40.59	0.204
VLBW rate (< 1500 g) (%)	(100/3611)2.77	(3/170)1.76	0.628
Birth weight discordance>25% (%)	(119/3320)3.58	(5/78)6.41	0.207
Percentage of boys (%)	(1997/3611)55.30	(100/170)58.82	0.367
obstetric outcomes			
Gestational hypertension (%)	(105/2104)4.99	(8/122)6.56	0.443
gestational diabetes mellitus (%)	(48/2104)2.28	(5/122)4.10	0.210
intrahepatic cholestasis of pregnancy (%)	(8/2104)0.38	(2/122)1.64	0.101
twin-twin transfusion syndrome (%)	(0/2104)0	(4/122)3.28	***p*** **< 0.001**
Neonatal deformities (%)	(24/3611)0.66	(1/170)0.59	1.000

## Discussion

Multiple pregnancies significantly increase the incidence of severe fetal and maternal complications. To avoid these risks, we advocate single pregnancy in order to achieve good obstetric outcomes. To ensure both pregnancy rates and obstetric outcomes, we recommend the number of transfer embryos should not exceed two cleavage embryos or one blastocyst in the first cycle. Still, multiple pregnancies are inevitable. There are many reasons for multiple pregnancy, such as maternal age, embryo quality, blastocyst transfer, laboratory environment, culture medium conditions, genes and genetic factors, zona pellucida operation, etc. Our previous study has confirmed that gestational age < 35 years old and blastocyst transfer are independent risk factors for monozygotic twinning (MZT) [6].

Multiple pregnancy can easily lead to gestational hypertension, gestational diabetes mellitus, intrahepatic cholestasis of pregnancy, anemia, premature rupture of membranes and premature delivery, low birth weight, and abnormal fetal development. Fetal reductions have emerged as a remedy to reduce the risks associated with multiple pregnancies. It is difficult to decide whether MFPR or not while considering the risk of abortion. The aim of this research was to compare the pregnancy and obstetric outcomes of different reduction tactics and expectant management in DCTA and TCTA pregnancies.

DCTA pregnancies have many associated risks, such as premature delivery, selective growth restriction, and fetal malformations. Monochorionic twins are associated with single placental bed vascular anastomoses, such as TTTS and selective intrauterine growth restriction [16, 17]. These adverse pregnancy and obstetric outcomes have led to search for a favorable fetal reduction strategy for reducing the occurrence of the aforementioned adverse events. However, although MFPR can reduce the premature delivery rate, the miscarriage rate may increase correspondingly. Therefore, no consensus exists on whether MFPR should be performed and the optimal number of fetal reductions in DCTA pregnancies [18]. Some research has shown that MFPR to singleton in DCTA may improve the pregnancy outcomes and positively alter gestational week, related to infant mortality and disability [9, 18–21].

A systematic review of different treatment strategies in DCTA suggested that expectant management is a reasonable option When survival rates are prioritized. Conversely, if minimizing the rate of severe premature delivery is the top priority, the best desirable choice is to reduce the number of fetuses [20]. The research of Chaveeva et al. supports the conclusion that embryo reduction increases the miscarriage rate but reduces the premature delivery rate in DCTA pregnancy [21]. Likewise, a smaller meta-analysis noted that the miscarriage rate of expectant management group were almost twice as likely as the MFPR group (8.1% vs. 4.4%) [22]. However, another small meta-analysis found no difference in the miscarriage rate at 24 weeks between multiple reduction group and expectant management group [23]. A larger meta-analysis by Zipori Y et al. found that reduction of triplet pregnancy to twins’ group had a lower rate of preterm birth before 32 or 28 weeks of gestation and maternal complications, such as gestational diabetes, gestational hypertensive disorders, need for antenatal hospitalization, and cesarean delivery rates and a higher rate of fetal birth weight compared with the expectant management group, and the miscarriage rate did not increase within 24 weeks of gestation. However, the rates of small for gestational age (SGA) births, overall survival are comparable between reduction group and expectant management group [24]. Our research found that the complete miscarriage rate was significantly reduced from 29.41 to 2.63% in DCTA reduction to singleton than DCTA-expectant management group. However, no difference was found in complete miscarriage rates between the TCTA reduction group and expectant management group. And in this study, the complete miscarriage rate after reduction was lower than previous addressed. This finding demonstrates that DCTA reduction to singleton is safe, and the miscarriage rate has not increased during the 6–8 weeks gestational period.

The timing of fetal reduction in multiple pregnancies has previously been studied in detail. The miscarriage rate after reduction in multiple pregnancies also varies at different times. A study by Evans showed that pregnancy loss rates were 5.4, 8.7, 6.8 and 9.1% when the timing of fetal reduction was 9–12 weeks, 13–18 weeks,19–24 weeks, and ≥ 25 weeks, respectively, but the data were not statistically different [25]. Another study showed that the miscarriage rates were 4.3 and 4.0% when MFPR to twin at 11–12 weeks and 13–14 weeks, respectively. The data are also not significantly different [26]. Haas et al. suggested that compared MFPR to twin with MFPR to singleton at 6–8 weeks gestation, the miscarriage rates before 24 weeks were 3.6 and 5.3%, respectively [14]. MFPR at 11–14^+ 6^ weeks had a lower rate of spontaneous abortion compared with 15–24^+ 6^ weeks’ gestation (6.5% vs. 14.9%, respectively) [27]. In this study, DCTA reduction to singleton achieved a lower miscarriage rate of 2.63% at 6–8 weeks’ gestation compared to DCTA-expectant management group.

DCTA reduction to twin group are composed of 18 underwent selective reduction of one fetuses of the monochorionic-diamniotic twin. Because of the vascular anastomosis between monochorionic twins [28]. when reducing one of the monochorionic twins, more caution should be undertaken while performing fetal reduction surgery, and the complete miscarriage rate of TCTA-expectant management was significantly lower than that of DCTA-expectant management in our study.

There were significant differences between the subgroups of participants who underwent MFPR to singleton compared to those who choose MFPR to twin or expectant management in DCTA and TCTA pregnancies, which shows that MFPR reduction to singleton improved pregnancy and obstetric outcomes by obviously reducing the risks of premature delivery and LBW, and obviously raising gestational week and average birth weight in DCTA and TCTA pregnancies. DCTA and TCTA reduction to singleton group had the lowest rate of preterm birth. In terms of obstetric complications, the DCTA reduction to singleton group had the lowest incidence of gestational diabetes. Similarly, other findings also showed that the triplet pregnancy to twins group had a lower incidence of gestational diabetes and preterm birth than the expectant management group [29]. Some researchers believe that the cause of preterm birth may be related to the relative lack of adequate uterine cavity and blood supply. Whereas the subdivided subgroups of TCTA had no significant difference in the incidence of gestational diabetes. This result may be related to the limited amount of data in the TCTA subgroup, and later studies can increase the amount of relevant data for further research. Thus, we didn’t advocate expectant management for DCTA pregnancy.

Potassium chloride injection is not recommended for monochorionic pregnancy because the remaining fetus can be embolized by the drug through vascular anastomosis in the common placenta [30]. However, the laser technique of intrafetal interstitia to remove one monochorionic twin can also imperil the remaining twin [31]. Chaveeva et al. reported 61 pregnant women with DCTA whose pregancies were reduced to dichorionic twin pregnancy by intrafetal laser ablation; although 3% of cases of miscarriage occurred after reduction, nearly half of the cases occurred within 2 weeks after reduction [15]. Other studies show that the mechanical method of intracardiac puncture and aspiration is an effective and feasible MFPR method for reducing adverse pregnancy outcomes, including those in monochorionic twin pregnancies [32–35]. Therefore, we adopted the mechanical method of intracardiac puncture and aspiration to reduce the fetus during the 6–8 weeks gestational period.

The miscarriage rate of DCTA-expectant management group was obviously higher than DCTA reduction to singleton group, although the mechanism of miscarriage is not clear. Some researchers think the relative lack of adequate uterine cavity and blood provision is related to spontaneous fetal reduction in multifetal pregnancy [36]. However, we also found that pregnancy loss occurs after embryo reduction in DCTA and TCTA pregnancies. Compared TCTA reduction to twin with DCTA reduction to twin group, TCTA reduction to twin group would obtain the proportion of two babies is significantly higher than that of DCTA reduction to twin group. This dramatically higher singleton survival rate and dramatically lower twin survival rate in DCTA pregnancy is consistent with the findings of Li et al. [33]. According to some studies, the related mechanism of miscarriage caused by fetal reduction in DCTA may be considered as follows: firstly, injuries and infections caused by fetal reduction surgery in cases where miscarriage occurred within 2 weeks of fetal reduction. Secondly, the necrotic embryonic placental tissue causing the inflammation reaction is reabsorbed, which could cause miscarriage several weeks or months after fetal reduction [30, 37, 38] Therefore, when considering reducing the complications of pregnancy and adverse obstetric outcomes and choosing to reduce fetuses to dichorionic twins in DCTA pregnancies, couples should be informed about the higher risk of pregnancy loss. There was no significant difference in cesarean section rate and the percentage of boy between the three subgroups in DCTA and TCTA pregnancies. The main reason why there is no difference in the rate of cesarean section may be due to human factors rather than medical needs.

Due to the unique characteristics of monochorionic twin pregnancies in terms of the placental structure, some studies conclude that monochorionic twins have dramatically worse outcomes than dichorionic twins [31, 39, 40]. This may be attributable to the complications associated with monochorionic twins, including TTTS, TAPS, and SIGR, which are detrimental to maternal and fetal health. Liu et al.’s study shows that the pregnancy and obstetric outcomes of DCTA-monochorionic twin pregnancies are relatively worse than those retaining a single fetus but without statistical difference. It is concluded that compared with reducing one fetus in monochorionic twins, reduction with a separate placenta might be an acceptable reduction strategy with a relatively lower miscarriage rate, despite the potential risks to monochorionic twins [19]. However, this research found that the complete miscarriage rate of the DCTA reduction to twin group is slightly higher than that of the DCTA reduction to singleton group, but there is no significant difference. Therefore, regarding the choice of DCTA to twin pregnancy, we must weigh the pros and cons and solicit the choice of couples, informing patients of the risks and benefits of reduction to one or two or expectant management.

This was a single-center retrospective comparative study, and some of the statistically insignificant results may be due to the limited number of patients in some subgroups. The Eligible patients are not randomly assigned to each group, so the results of the study may have some deviations. Due to different wishes and internal factors of the family, and ethical considerations, some couples may choose to undergo MFPR or not, and this research is unlikely to be suitable for randomized controlled trials. Some of the data were collected through telephonic interviews with women who had been pregnant many years before thereby the data could be prone to recall bias. Some of the strengths of our study include the relatively abundant reduction data, an extended research time frame, strict inclusion criteria, and detailed statistical methods. All reduction operations were performed by several highly skilled doctors in our center, thereby preventing significant differences in surgical results.

## Conclusions

MFPR could improve pregnancy and obstetric outcomes for DCTA and TCTA pregnancies, and MFPR to single fetus could achieve a longer gestational week and higher average birth weight. It seems that dichorionic twins have better pregnancy outcomes than monochorionic twins. For DCTA pregnancy, it is highly recommended to reduce fetus to single for the best pregnancy and obstetric results. Fetal reduction is simply a remedy to reduce the risks related to multifetal pregnancy. With the rapid progress of embryo culture technology in vitro and high embryo implantation rate in China, single blastocyst transplant has been encouraged in more and more Reproductive Medicine Centers to prevent multifetal pregnancy.

## Data Availability

The datasets generated and/or analysed during the current study are not publicly available due the containing information that could compromise research participant but are available from the corresponding author on reasonable request.

## Abbreviations

MFPR: Multifetal pregnancy reduction
DCTA: Dichorionic triamniotic triplet pregnancy
TCTA: Trichorionic triamniotic triplet pregnancy
ICSI: Intracytoplasmic sperm injection
IVF: In vitro fertilization
ART: Assisted reproductive technology
MC-DA: Monochorionic-diamniotic
DCDA: Dichorionic-diamniotic
TTTS: Twin-to-twin transfusion syndrome
SIGR: Selective intrauterine growth restriction
TAPS: Twin anemia-polycythemia sequence
ET: Embryo transplant
FET: Frozen embryo transplant
BMI: Body mass index
LBW: Low birth weight
VLBW: Very low birth weight
MZT: Monozygotic twinning
SGA: Small for gestational age
